# Evidence of Antimicrobial Resistance and Presence of Pathogenicity Genes in *Yersinia enterocolitica* Isolate from Wild Boars

**DOI:** 10.3390/pathogens10040398

**Published:** 2021-03-27

**Authors:** Paola Modesto, Chiara Grazia De Ciucis, Walter Vencia, Maria Concetta Pugliano, Walter Mignone, Enrica Berio, Chiara Masotti, Carlo Ercolini, Laura Serracca, Tiziana Andreoli, Monica Dellepiane, Daniela Adriano, Simona Zoppi, Daniela Meloni, Elisabetta Razzuoli

**Affiliations:** 1 Istituto Zooprofilattico Sperimentale del Piemonte, Liguria e Valle d’Aosta, Piazza Borgo Pila 39/24, 16129 Genoa, Italy; walter.vencia@gmail.com (W.V.); mariaconcetta.pugliano@izsto.it (M.C.P.); 2 Istituto Zooprofilattico Sperimentale del Piemonte, Liguria e Valle d’Aosta, Via Nizza 4, 18100 Imperia, Italy; Walter.Mignone@izsto.it (W.M.); enrica.berio@izsto.it (E.B.); 3 Istituto Zooprofilattico Sperimentale del Piemonte, Liguria e Valle d’Aosta, Via degliStagnoni 96, 19100 La Spezia, Italy; chiara.masotti@izsto.it (C.M.); carlo.ercolini@izsto.it (C.E.); laura.serracca@izsto.it (L.S.); 4 Istituto Zooprofilattico Sperimentale del Piemonte, Liguria e Valle d’Aosta, Via Martiri 6, 17056 Savona, Italy; tiziana.andreoli@izsto.it (T.A.); monica.dellepiane@izsto.it (M.D.); 5 Istituto Zooprofilattico Sperimentale del Piemonte, Liguria e Valle d’Aosta, Via Bologna 148, 10154 Turin, Italy; daniela.adriano@izsto.it (D.A.); simona.zoppi@izsto.it (S.Z.); daniela.meloni@izsto.it (D.M.)

**Keywords:** *Yersinia enterocolitica*, wild boar, pathogenicity, biotypes, antibiotic-resistance

## Abstract

*Yersinia enterocolitica* (*Ye*) is a very important zoonosis andwild boars play a pivotal role in its transmission. In the last decade, the wild boar population has undergone a strong increase that haspushed them towards urbanized areas, facilitating the human–wildlife interface and the spread of infectious diseases from wildlife to domestic animals and humans. Therefore, it is important to know the serotype, antimicrobial resistance and presence of pathogenicity genes of *Yersinia enterocolitica* (*Ye*) isolated in species. From 2013 to 2018, we analyzed the liver of 4890 wild boars hunted in Liguria region; we isolated and serotyped 126 *Ye* positive samples. A decisive role in the pathogenicity is given by the presence of virulence genes; in *Ye* isolated we found *ystB* (~70%), *ymoA* (45.2%), *ail* (43.6%) and *ystA* (~20%). Moreover, we evaluated the susceptibility at various antimicrobic agents (Ampicillin, Chloramphenicol, Enrofloxacin, Gentamicin, Kanamycin, Trimethoprim–Sulfamethoxazole, Sulfisoxazole, Ceftiofur and Tetracycline). The antibiotic resistance was analyzed, and we found a time-dependent increase. It is important to shed light on the role of the wild boars as a reserve of potentially dangerous diseases for humans, and also on the antibiotic resistance that represents a public health problem.

## 1. Introduction

*Yersinia enterocolitica* (*Ye*) are zoonotic psychrotropic bacteria, which cause acute gastroenteritis and, occasionally, more serious diseases in humans. Yersiniosis was the fourth zoonosis reported in humans in 2018, with 6699 confirmed cases reported in Europe (EU). The trend of human cases was stable in 2014–2018, confirming the trend in 2019; however, in 2017 the number of confirmed cases was 2.8% lower than in 2016, and represented the lowest recorded rate in the last 5 years [1,2]. *Ye* was the most common species reported to be isolated from human cases, in food and in animals. The more widespread serotypes in human yersiniosis were O:3, followed by O:9 and O:8. Moreover, the biotype prevalent in 2016 was biotype 4, followed by biotype 2 and 3. In 2018, biotypes and serotypes of *Ye* were rarely reported [1]. *Ye* species forms a heterogeneous group of non-pathogenic and pathogenic strains. *Ye* comprises six biotypes: 1A, 1B, 2, 3, 4 and 5, based on metabolic differences which are further classified into numerous serotypes [3,4,5,6]. Biotype 1A is often recognized to be avirulent; however, some biotype 1A strains can be a cause of gastrointestinal symptoms and sporadic extraintestinal infections [3,7,8,9]. The virulence of the strains belonging to biotypes 1B and 2–5 depends on the presence of both chromosomal and plasmid-borne genes [10]. The presence of a high pathogenicity island (HPI) encoding for the yersinia bactin siderophore system determines the high pathogenicity of biotype 1B strains infection in the mouse model, while biotypes 2–5 constitute low–moderate pathogenic lineages [4,5,11,12,13]. Only few serotypes are known to be injurious to humans and were associated with different clinical manifestations. Bioserotypes 1B/O:8, 2/O:5,27, 2/O:9, 3/O:3 and 4/O:3 (in order of increasing frequency) are the most frequently isolated pathogenic strains in Europe [14,15,16]. The pathogenicity of *Ye* is often associated with chromosomal virulence genes that comprise *Attachment and invasion locus* (*ail*), *Invasin* (*inv*), *Mucoid Yersinia factor* (*myf*), *Host-responsive element* (*hreP*) and *Yersinia stable toxin* (*yst*) [17,18]. Additionally, *Yersinia-modulating protein*(*ymoA*) is an important chromosomal gene encoding for the YmoA protein, which negatively regulates the expression of various genes; it inhibits the expression of *inv* and *Yersinia stable toxin A* (*ystA*) [19].Regarding virulence plasmid genes (*pYV*), there are known genes: *Adhesin A* (*yadA*), whose product is involved in autoagglutination, serum resistance and adhesion [20]; *Transcriptional regulator* (*virF*), which encodes transcriptional activators of the yop regulon [21], and is therefore fundamental for the type-III secretion system. Biotype 1A is recognized as non-pathogenic, since they do not have *pYV* plasmid and some chromosomal virulence genes, e.g., *ystA* and *myfA* [9]. Although *inv* is present, it seems to be non-functional in most 1A strains [22]. However, the 1A strains carry other virulence genes, such as *ystB* and *hreP*, and some biotype 1A strains that were involved in human infections [23]. The epidemiology of the infection and the distribution of serotypes need to be further understood [2,24]. It is known that infection occurs after the consumption of poorly cooked pork meat or vegetables, and healthy pigs are the principal reservoir of *Ye* [25,26]. Indeed, the pathogen is often isolated from the tonsils, intestines or faeces of swine. Furthermore, *Ye* can be isolated from soil, water, and the environment, in which it is able to survive for a long period [27]. Moreover, there is evidence of the link between pigs, pork carcasses, wild boars and associated products [28,29,30,31]. Indeed, *Ye* are sometime isolated in wild boars, and recent papers showed a prevalence between 3.5% and11% in European wild boars [31,32,33]. However, data on their pathogenicity and antimicrobial resistance are still lacking. This is particularly concerning in highly urbanized areas like Liguria region (Italy), where the increased human–wildlife interface facilitates the spread of infectious diseases from wildlife to domestic animals and humans. The aim of our study was to investigate the presence and thedetection of biotypes of *Ye* in the wild boars hunted in Liguria region from 2013 to 2018, and to evaluate the presence of chromosomic genes of pathogenicity (GoP) and *Ye* antimicrobial resistance.

## 2. Results

### 2.1. Y. enterocolitica Isolation, Biotyping and Serotyping

*Ye* was isolated in 126 samples (2.6%) out ofthe 4890 analyzed; all strains were isolated from the liver of wild boars hunted in province of Genoa (108/126) or La Spezia (18/126). Each strain isolated from positive samples was bio-serotyped (BT): the most common biotype was 1A (*n* = 117, 92.9%), followed by 1B (*n* = 8, 6.3%) and 2 (*n* = 1, 0.8%).

**Figure 1:**
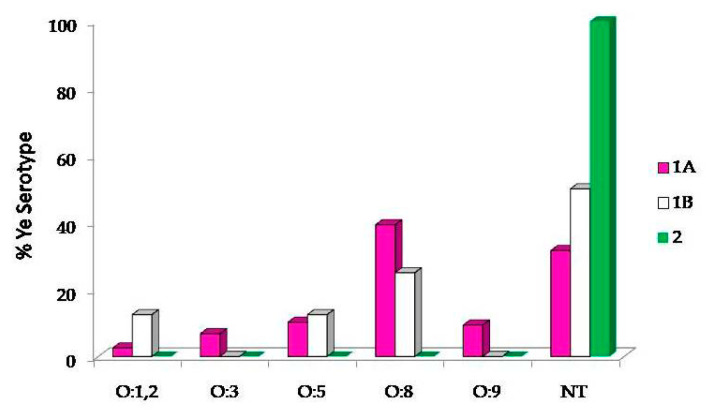
Percentage of *Yersinia enterocolitica* (*Ye*) serotypes distribution. In biotypes 1A and 1B, the most frequent serotype was O:8, followed by O:5. The biotype 2 was represented by a single not-typable sample. However, in all biotypes (1A, 1B and 2) an elevated percentage of serotypes was found not-typable (31.69%, 50% and 100%, respectively).

We observed the circulation of several serotypes (ST): O:1,2; O:3; O:5; O:8 and O:9 (Figure 1). In detail, 48 strains (38.1%) were ST O:8, 13 strains (10.3%) were ST O:5, 11 strains (8.7%) were ST O:9, 8 strains (6.3%) were ST O:3 and 4 strains (3.2%) were ST O:1,2 (Figure 1). A large amount of isolated strains (42/126, 33.3%) were not-typable (NT) using the available sera. Focusing on the serotypes detected for the 1A biotype isolates, the most common BT was 1A/O:8 (46/126, 36.5%), followed by BT 1A/O:5 (12/126, 9.5%), BT 1A/O:9 (11/126, 8.7%), 1A/O:3 (8/126, 6.3 %) and 1A/O:1,2 (3/126, 2.4%). Concerning the 1B BT (the second by frequency, 8/126), half of the samples were not serotypable, while the other half were classified as O:5 (12.5% of 1B), O:8 (25% of 1B) and O:1,2 (12.5% of 1B). The only, isolate of the BT 2 was NT (Figure 1).

### 2.2. Presence of Chromosomic Genes of Pathogenicity

Table 1 reports the results about the presence of chromosomic genes of pathogenicity, and in Table 2 the pathogenicity genes’ percentages are depicted. *ystB* was found in the 70%, *ail* in the 44% and *ymoA* in the 45% of the strains. Some positive results werealso obtained also for *ystA* (20%), *myfA* (12%) and *inv* (8%).

Individually considered, in the biotype 1A, *ystB* was identified as the more frequent gene of pathogenicity, as more than half of the strains were positive (68.4%). Additionally, *ail* and *ymoA* were detected with a frequency of 45.3% and 44.4%, respectively, while, for the other genes analysed (*ystA*, *myfA* and *inv*), the percentages were about 20%, 12% and 8.5%, respectively. The biotype 1B showed the highest presence of the *ystB* gene of pathogenicity (87.5%), *ymoA* was present in 50%*, ail* in 25%, *ystA* and *myfA* were equally present in 12.5% of the strains. None of the strains were positive for *inv* gene. The biotype 2, isolated in theliver of one wild boar, was characterized by the presence of *ystA*, *ystB* and *ymoA*.

### 2.3. Antimicrobial Susceptibility

All *Ye* isolates were tested and 61.9% (*n* = 78) showed resistance at least to one drug: 85.71% of the microorganism were resistant to Ampicillin, 23.8% to Triple-Sulfa and Sulfisoxazole, and 7.14% to Ceftiofur. Antimicrobial resistance to Chloramphenicol and Enrofloxacin was not found; moreover, the strains had shown very low resistance against Streptomycin and Tetracycline (0.79%; Table 3). An increasing antibiotic resistance trend towards Ampicillin, Triple-Sulfa, Sulfisoxazole and Ceftiofur was shown (Table 4). Moreover, concerning multiples’ resistances, we observed that 12 strains were resistant to two antibiotics, 14 to three antibiotics, 5 strains were resistant to four antibiotics and 9 to five antibiotics.

The results showed an increasing frequency of the multi-drug resistance (MRS) in the strains isolated from 2013 to 2018: 9% 2014, 30% 2015, 38% 2016 and 40% 2017, respectively (Table 5).

In particular, the analyses demonstrated an increase in resistance toward the association of different pairs of antibiotics (Table 6). Ampicillin and Ceftiofur resistance was only seen in the seasons 2015–2016 and 2016–2017 (6.6% and 13% respectively). Ampicillin and Triple-Sulfa resistance was 13.3% 2015–2016, 35.1% 2016–2017 and 35% 2017–2018, respectively. Ampicillin and Sulfisoxazole resistancewas 4.5% 2014–2015, 16.6% 2015–2016, 29.6% 2016–2017, and 35% 2017–2018, respectively (Table 6).

## 3. Discussion

*Yersinia enterocolitica* is a zoonotic pathogen which causes acute gastroenteritis and, occasionally, more serious diseases in humans [27]. Today, there is no harmonized surveillance of *Ye* in the EU: recorded data are not comparable between member states and extreme caution is needed when interpreting results at the EU level; nevertheless, yersiniosis is the fourth most reported zoonosis in the EU [1]. There was a decreasing trend in reported confirmed human cases of yersiniosis in the EU/EEA from 2008 to 2018, but the trend did not show any significant increase or decrease in the past 7 years (2013–2019). The highest country-specific notification rates were observed in northeastern European member states. *Ye* was the most common reported pathogens in these states, and it was identified in 11food-borne outbreaks. The most common bioserotype was 4/O:3, followed by 2/O:9 and 2/O:5,27. Very few European member states reported food and animal data on *Yersinia* occurrence or prevalence in 2017; indeed, reporting this kind of data is not mandatory. These scarce data preclude meaningful observations at the EU level. According to the last EFSA report, *Yersinia* has been isolated mainly in pork fresh meat (8.3%), in meat products from sheep (16%), beef cattle (6.3%) and in living animals (pigs 4.4 %, other animals 3.5%) [1].

In wildlife, European authors reported a prevalence between 33.3% and 1.3%, in Spain and Poland, respectively [34,35]. Other studies have highlighted the influence of seasonality on the prevalence; these authors reported a prevalence of 17.1% in Germany and 20% in Sweden, with the highest values recorded in cold seasons (winter and spring) [31,36].

In our study, *Ye* was isolated on 2.9% of animals; thesedata are in accordance with the 3.5% prevalence reported in the EFSA report of the 2014 [37], and with the study of Bancerz-Kisiel [35]. Despite the low prevalence, it is worth remembering that the wild boar population increased significantly in the last century, both in European and Italian territories. As a result, the species hasspread to new areas and contact with humans and livestock increased simultaneously with the risk to public safety [35]. In this condition, it may be useful to characterize the strains of the wild boar populations in order to know their serotype, biotype and, above all, the pathogenic potential.

*Ye* is classified by the heat-resistant somatic antigen O (seventy serotypes described) and by the biotype. To date six biotypes are known: 1A, 1B, 2, 3, 4, 5. Strains O:3 and O:9 are often isolated from swine that are considered the main reservoir, and strains O:8 are isolated from water, vegetables and dairy food. In our study, 33.3% of the strains was not characterized: 38.1% was O:8 serotype and10.3% was O:5 serotype, which wereboth associated to human gastroenteritis cases [38]. Our results differ from data reported by Kamińs kaand Sadkowska-Todys [39], which highlighted the circulation of O:3 (88%), O:8 (6.9%) and O:9 (5.2%) strains. These differences could be due to a non-correlation between serotypes and geographical distribution [40]. Reports on the *Ye* presence in wild boars are rare, and the epidemiological link between wild boars and domestic pigs is still unknown [31,41]. In our study, the higher frequency of serotype O:8 suggests that, in our region, the major source of *Ye* in wild boars is anthropogenic; moreover, we can speculate that water could be the link between humans and wild boars [42]. Indeed, most *Ye* isolates in water belong to non-biotype 1A or to *Ye*-like bacteria [42].

In regard to the biotypes, the biotype 4 (serotype O:3) and 2 (serotype O:9) are more frequently associated with human yersiniosis: strains 1A were isolated from environment, foods, and human and animal faeces [25]. 1A strains are not supposed to be pathogens and are not presumed to represent a risk to public safety [19]. Our results highlight the high prevalence of the 1A biotype; indeed, we detected 117 (92.9%) strains of the biotype 1A, 8 strains of the biotype 1B and 1 strain of the biotype 2. Among the European states, Bancerz-Kisiel2016 [43], have reported a 1A strains prevalence of 15.4% in Poland.In Italy, there are no data other than ours on wild boars’ *Ye* 1A prevalence. However, *Ye* detection in swine has been reported by Bonardi [44]. In particular, 11.2% of samples (19 amygdales out of 170) obtained from 19 different farms, located in the province of Mantua, Brescia, Reggio Emilia, Verona, Parma and Cuneo were found to be positive. Most of them belonged to serogroup O:3 biotype 4 (13/19, 68.4%), while 15.8% (3/19) belonged to bio-serotype 1A/O:8, 10.5% (2/19) at bio-serotype 1A/O:5 and only 5.2% (1/19) at bio-serotype 4/O:8. The distribution of genes associated with virulence appeared to vary, with a prevalence, within the bio-serotype 4/O:3, of positive strains for both *ail* and *ystA* genes, and positive strains for the three sequences *yadA*, *ail* and *ystA.*

Some studies suggested that few 1A strains may be the cause of intestinal infection. According to Liang and colleagues [45], the 1A isolates lack the *pYV* plasmid and are therefore considered non-virulent. Although, some studies have demonstrated *Ye* 1A ability to invade the epithelial cells and to cause symptomatology indistinguishable from that caused by pathogenic biotypes (1B, 2–5) [46]. Moreover, our recent study demonstrated the ability of different strains of *Ye* 1A to adhere to and penetrate enterocytes, causing an innate immune response characterized by a strong pro-inflammatory response [47].

In the present study, to evaluate the pathogenic potential of the isolates, we assessed the presence of chromosomic genes of virulence and 69.8% of the strains was positive for *ystB*, *45.2%* positivefor *ymoA*, 43.6% was positive for *ail* and 19.8% was positive for *ystA*. A small percentage of the isolates was positive for *myfA* and *inv* (11.9 and 7.9%, respectively). In our study, we outlined a major presence of *ail* and *yst* genes with respect to the study conducted by Younis and coworkers in Egypt [48]. Our results showed the presence of *ystB* both in 1A and 1B biotypes; therefore, on the basis of Liang et al.’sassumptions [45], it could be speculated that both 1A and 1B biotype strains are pathogenic. More in vitro and in vivo investigations are needed to assess that event.

Data regarding the strains’ pathogenicity are of high interest if correlated to antimicrobial resistance or tolerance to biocides. In this respect, a recent study demonstrated the ability of *Ye* to acquire tolerance to biocides and to increase its antibiotic resistance after exposure to sub-MICs of such disinfectants [49].

In our study, 61.9% of the isolates showed antimicrobial resistance, with an increase from 2013 to 2017. Similar results were obtained in Egypt [48]. In 31 Bavarian farms from 2000 to 2004, a study on *Ye* strains showed that 77% of 4/O:3 strains were sensitive to 14 antimicrobial drugs [50]. A sporadic resistance was observed against Amoxicillin and Clavulanic acid (5%), Streptomycin (9%), Sulfamethoxazole (9%) and Tetracycline (1%). Similar levels in swine isolates resistance were found in Switzerland and Brasil [51,52].

According to Italian studies, a large spread of multi-drug-resistant isolates has been detected, and strains resistant to three or more antimicrobial drugs were detected in 91% of the isolates 4/O:3. The resistance against Chloramphenicol was detected in all the strains [53]. In a study conducted in China, high levels of antimicrobial resistance were found on *Ye* 4/O:3 strains: Sulfonamide (91%), Streptomycin (64%) and Chloramphenicol (55%) [45]. Bhaduri [54] has tested the resistance profile in isolates both *pYV* positive and negative and reported a high resistance to Tetracycline in USA (27%); Simonova [50] has reported resistance to the Nalidixic acid (4%) and to Chloramphenicol (4%).In the Czech Republic, the presence of *pYV* gene has not been associated to the profile of resistance, as described by Bhaduri [54]; moreover, no correlation has been found between the different *Ye* 4/O:3 genotypes and the models of antimicrobial resistance [55]. It is known that *Ye* can produce two chromosomal β-lactamases, BlaA and BlaB. The latter can induce a broad spectrum cephlaosporinase that has a different activity in *Ye* biotypes; in particular, a recent study suggest that BlaB is more inducible in biotypes 2 and 4, than in biotypes 1 A and 1B [55,56].

The possible causes of the development of antimicrobial resistance are: (i) massive treatments carried out for the prophylaxis of bacterial infections; (ii) inaccurate dosage of antimicrobial drugs; (iii) inadequateduration of the treatments; (iv) usage of antimicrobial as growth promoters.

The detection of antimicrobial resistance in strains isolated in wild boar could be associated to two main factors: transfer of the antimicrobial resistance occurring, by means of plasmids, between strains of the same or different species (hosted in both swine and wild boar) [57] and the rapid increase in the wild boar population, which is causing more frequent contacts with domestic livestock (mainly pigs). Whatever the cause, the presence of antimicrobial-resistant strains isolated in wildlife samples is an important aspect to consider due to its impact on public health [58].

## 4. Materials and Methods

### 4.1. Samples Collection and Y. enterocoliticaIsolation

A total of 4890 liver samples were collected from wild boars hunted in Liguria during five hunting seasons between September 2013 and January 2018 and were tested for the presence of *Ye* by the standardized ISO 10273:2003 method. Briefly, after 5 days of incubation in Phosphate Buffered Saline (PBS, AMRESCO, VWR Int., Milan, Italy, cat 3546423) a 25 ± 1 °C; 0.1 mL of broth was seeded in cefsulodin-irgasan-novobiocin (CIN) agar plates (Oxoid, Nürtingen, Germany) and incubated 24–48 h at 30 ± 1 °C. Then, CIN plates were checked for characteristic colonies. Typical colonies, lactose-negative and urease-positive, were submitted to biochemical identification with API^®^ 20 E system (bioMérieux, Marcy l’Etoile, France).

### 4.2. Y. enterocolitica Biotypingand Serotyping

All *Ye* strains isolated were biotyped and serotyped according to procedure ISO10273-2003. In order to define the serogroups of our isolates, the commercially available agglutination tests for O:3, O:5, O:1.2, O:8 and O:9 were used. First, an autoagglutination test (using saline solution) was performed for each isolate, then a single colony was mixed with a polyvalent serum on a glass slide and swung for 30 s. When the agglutination was observed (positive reaction), in order to identify the serogroup, the procedure was repeated using a monovalent serum.

### 4.3. Real Time PCR for Chromosomic Gene of Pathogenicity 

Each *Ye* strain isolated was checked forthe presence of six chromosomic virulence genes. Attachment and invasion locus (*Ail*), invasin (*inv*), Yersinia stable toxin A (*ystA*), Yersinia stable toxin B (*ystB*), mucoid Yersinia factor (*myfA*) and Yersinia modulator *(ymoA*) were investigated using a primer set described in previous studies (Table 7) [23,45,59,60,61,62,63]. DNA was extracted from pure colonies using QIAmp DNA mini kit^®^ (Qiagen, Milan, Italy). Two microlitres of the DNA (concentration *n* = 25 ± 5.4 ng/μL) wereused as a template for Ye Real-Time PCR and added to 18 μL of mastermix contains 10 μL of iQ™ SYBR Green Supermix (Bio-Rad, Milan, Italy) to 0.2 μM of forward and reverse primers and 4 μl of H_2_O, a negative and positive control have been added to each run. The Real-Time PCR amplification was run on a CFX96 Real-Time System (Bio-Rad) following the PCR thermal protocols previous described [60]. After the amplification protocol, samples showing a threshold cycle (Cq) under 37 and a specific melting temperature (Tm) were considered as positive.

### 4.4. Antimicrobial Susceptibility Test

The Kirby–Bauer disc diffusion test was performed following the Clinical and Laboratory Standard Institute (CLSI) guidelines (M02–A11, 2012), using Mueller-Hinton agar plates (Microbiol, Italy). The antimicrobials and used quantities (μg) were: Ampicillin (A, 10; Sigma Aldrich, Saint Louis, MO, USA), Chloramphenicol (C, 30; Sigma Aldrich, Saint Louis, MO, USA), Enrofloxacin (ENR, 5;Thermofisher Scientific, Milan, Italy), Gentamicin (G, 10;Thermofisher Scientific, Milan, Italy), Kanamycin (K, 30; Sigma Aldrich, Saint Louis, MO, USA), Trimethoprim–Sulfamethoxazole (SXT, 1.25/23.75; Sigma Aldrich, Saint Louis, MO, USA), Sulfisoxazole (ST, 300; Thermofisher, Thermofisher Scientific, Milan, Italy), Ceftiofur (EFT, 30; Thermofisher Scientific, Milan, Italy) and Tetracycline (T, 30; Thermofisher Scientific, Milan, Italy). Data were analyzed following the Clinical and laboratory Standard Institute (CLSI) guidelines instructions (Table 2A Enterobacteriaceae M02 and M07, M100-S25, 2015).

## 5. Conclusions

The obtained data showed the circulation of *Ye* in Liguria region, with prevalence rates similar to those reported in the EFSA reports. Furthermore, the isolated strains show many of the pathogenicity genes under study, suggesting a pathogenetic potential even in microorganisms belonging to the 1A biotype. This hypothesis wasfurther investigated at the IZSPLV laboratories where host–pathogen interaction was evaluated in terms of modulation of the innate immune response, and penetration into enterocytes by means of an in vitro model of porcine enterocytes (IPEC-J2).

Furthermore, our data highlight the need for a correct handling of the wild pork meat, which is often consumedundercooked by hunters. Other concerns rise because theever-increasing phenomenon of the presence of multiple antibiotic resistances represents a serious risk to public health. With regard to this aspect, the study highlighted the need to implement training plans, also aimed at the population, that raise awareness of appropriate drug management.

## Figures and Tables

**Table 1:** Distribution of virulence genes among *Y*. *enterocolitica* isolates. Not-typable (NT). +: virulence genes positive.

Bio/Serotype (Number)	Virulence Genes					
	*ail*	*ystA*	*ystB*	*inv*	*ymoA*	*myfA*
1A/O:8 (46)	+(31/126)	+(8/126)	+(36/126)	+(5/126)	+(27/126)	+(8/126)
1A/O:5 (12)	+(6/126)	+(1/126)	+(7/126)	+(2/126)	+(6/126)	+(2/126)
1A/O:9 (11)	+(9/126)	+(5/126)	+(10/126)	+(1/126)	+(9/126)	+(2/126)
1A/O:3 (8)	+(3/126)	+(3/126)	+(5/126)	(0/126)	+(4/126)	(0/126)
1A/O:1,2 (3)	(0/126)	(0/126)	+(1/126)	(0/126)	(0/126)	(0/126)
1A/NT (37)	+(4/126)	+(6/126)	+(21/126)	+(2/126)	+(6/126)	+(2/126)
1B/O:5 (1)	+(0/126)	+(1/126)	+(1/126)	(0/126)	+(1/126)	(0/126)
1B/O:8 (2)	+(1/126)	(0/126)	+(1/126)	(0/126)	+(1/126)	(0/126)
1B/O:1.2 (1)	(0/126)	(0/126)	+(1/126)	(0/126)	+(1/126)	(0/126)
1B/NT (4)	+(1/126)	(0/126)	+(4/126)	(0/126)	+(1/126)	+(1/126)
2/NT (1)	(0/126)	+(1/126)	+(1/126)	(0/126)	+(1/126)	(0/126)

**Table 2:** Percentage of pathogenicity genes in bio-serotyped (BT) 1A, 1B and 2 isolates.

Pathogenicity Genes						
	*ail*	*ystA*	*ystB*	*inv*	ymoA	myfA
Total	43.6	19.8	69.8	7.9	45.2	11.9
1B	42.06	18.25	63.49	7.9	41.27	11.11
1A	1.58	0.79	5.55	0	3.17	0.79
2	0	0.79	0.79	0	0.79	0

**Table 3:** Percentage of antibiotic resistance showed by *Ye* isolates analyzed from 2013 to 2018.

Antibiotic	Percentage of Antibiotic Resistance
Chloramphenicol	0
Enrofloxacin	0
Gentamycin	1.58
Kanamycin	1.58
Streptomycin	0.79
Sulfamethoxazole Trimethoprim	3.17
Tetracycline	0.79

**Table 4:** Percentage of antibiotic resistance to the reported drugs during the year considered in the study.

Antibiotic	Percentage of Antibiotic Resistance 2013–2018			
	2014	2015	2016	2017
Ampicillin	21	26	43	18
Ceftiofur	0	6.6	12.9	0
Sulfisoxazole	4.5	20	29.6	35
Triple-Sulfa	0	13.3	35.1	35

**Table 5:** Percentage of strains showing antibiotic multi-resistance (MRS) increase ina time-dependent manner.

Years	MRS Percentage
2014	9.5
2015	30
2016	38.1
2017	40

**Table 6:** Percentage of MRS in *Ye* isolates considering pair of drugs.

Drugs	2014	2015	2016	2017
Ampicillin + Ceftiofur	0	6.6	13	0
Ampicillin + Triple-Sulfa	0	13.3	35.1	35
Ampicillin + Sulfisoxazole	4.5	16.6	39.6	35

**Table 7:** Primer Set for Real-Time Polymerase Chain Reaction Amplification.

Gene	Primer		Product Length (bp)	Accession Number	Source
*inv*	Forward	TGCCTTGGTATGACTCTGCTTCA	1144	X53368	23
	Reverse	AGCGCACCATTACTGGTGGTTAT		Z48169	
*myfA*	Forward	CAGATACACCTGCCTTCCATCT	271	Z21953	61
	Reverse	CTCGACATATTCCTCAACACGC			
*ymoA*	Forward	GACTTTTCTCAGGGGAATAC	329	X58058	62
	Reverse	GCTCAACGTTGTGTGTCT		AY387659	
*ail*	Forward	TAATGTGTACGCTGCGAG	54	JX972143	45
	Reverse	GACGTCTTACTTGCACTG		JQ665437	
*ystA*	Forward	ATCGACACCAATAACCGCTGAG	78	X65999	63
	Reverse	CCAATCACTACTGACTTCGGCT		X65999	
*ystB*	Forward	GTACATTAGGCCAAGAGACG	145	KM253278	63
	Reverse	GCAACATACCTCACAACACC		KM253279	

## Data Availability

Data is contained within the article.
